# Unveiling the antimicrobial and antibiofilm potential of biosurfactant produced by newly isolated *Lactiplantibacillus plantarum* strain 1625

**DOI:** 10.3389/fmicb.2024.1459388

**Published:** 2024-09-10

**Authors:** Babita Thakur, Sukhminderjit Kaur, Vagish Dwibedi, Ghadeer M. Albadrani, Muath Q. Al-Ghadi, Mohamed M. Abdel-Daim

**Affiliations:** ^1^Department of Biotechnology, University Institute of Biotechnology, Chandigarh University, Mohali, Punjab, India; ^2^Department of Biology, College of Science, Princess Nourah bint Abdulrahman University, Riyadh, Saudi Arabia; ^3^Department of Zoology, College of Science, King Saud University, Riyadh, Saudi Arabia; ^4^Department of Pharmaceutical Sciences, Pharmacy Program, Batterjee Medical College, Jeddah, Saudi Arabia; ^5^Department of Pharmacology, Faculty of Veterinary Medicine, Suez Canal University, Ismailia, Egypt

**Keywords:** probiotics, biofilm, biosurfactants, lactic acid bacteria, response surface methodology, antibacterial, anti-biofilm

## Abstract

The present study aimed to characterize the biosurfactants synthesized by lactic acid bacteria (LAB) obtained from fermented foods, optimize the conditions for increasing the yield of biosurfactants and explore their antimicrobial and antibiofilm potential. Out of the 26 LAB isolates, isolate BS2 showed the highest biosurfactant production as indicated in the oil displacement test, drop collapse and emulsification activity. BS2 was identified as *Lactiplantibacillus plantarum* 1625 using 16S-rRNA gene sequencing and phylogenetic analysis. The biosurfactant produced by BS2 was identified as an anionic glycol-lipo-proteins by employing Fourier Transform Infrared Spectroscopy (FTIR) and Gas Chromatography–Mass Spectrometry (GC–MS) analysis. The biosurfactants produced by *L. plantarum* 1625 demonstrated strong antibacterial and antibiofilm characteristics against pathogenic strains such as *Staphylococcus aureus* MTCC 1049, *Escherichia coli* MTCC 1587, and *Pseudomonas putida* MTCC 1655. The minimal inhibition concentration value of antibacterial activity was found to be 0.1 mg/mL with the inhibition percentage ranging from 90 to 95%. Further, the effect of temperature, pH, and substrate composition on biosurfactant production was also studied to enhance it production using the Box–Behnken Design approach of Response surface methodology (RSM). Application of biosurfactant led to a considerable decrease in biofilm-forming harmful bacteria, as proven by scanning electron microscopy analysis. The results highlight the potential uses of biosurfactants in distinct industries, and biotechnological contexts, especially in the creation of new antimicrobial and antibiofilm agents.

## 1 Introduction

The escalating threat posed by antibiotic-resistant microorganisms is a global health concern, necessitating the urgent development of novel antimicrobial and antibiofilm strategies (Devi et al., 2018). Biofilms, which are intricate bacterial populations enclosed in a protective matrix, are infamous for being resistant to medicines. This resistance makes infections challenging to cure, increasing the risk of prolonged sickness and higher medical expenses. Frequently, these biofilms accumulate on surfaces and medical equipment, which can lead to difficult-to-cure persistent illnesses. The increasing appearance of resistance mechanisms in many infections is causing antibiotics to lose their potency. Thus, it is crucial to explore distinct biological alternatives that are safe, effective, and able to reduce or avoid bacterial infections (Carolus et al., 2019). Biosurfactants, amphiphilic molecules synthesized by various microorganisms, have attracted significant interest as potential alternatives to conventional antibiotics. These molecules are surface-active and exhibit a variety of biological actions, such as immune-modulatory, antiviral, anticancer, and antibacterial qualities (Karnwal et al., 2023). Biosurfactants are also desirable for industrial and biomedical applications as they are often non-toxic, biodegradable, and ecologically benign (Khani et al., 2024). They are critical in mitigating ecological and human health concerns and aligning with sustainable solutions because biosurfactant (BS) plays an important role in dispersal, emulsification, mobilization, solubilization, surface tension reduction and foam formation (Zahed et al., 2022; Selva Filho et al., 2023). BS production methods usually include microbial fermentation and renewable resources lessening the reliance on petrochemicals and carbon footprint (Geys et al., 2014).

Even though the synthesis of BS by different species of bacteria has been extensively studied, a significant fraction of these strains are still underutilized because of their poor production rates. LAB which is commonly referred to as “probiotics” can produce BS which are safe to utilize in a range of pharmaceutical and biological applications due to their low toxicity, intrinsic biocompatibility and their positive effects on human health (Manga et al., 2021; Garcias-Bonet et al., 2023). Fermented foods, traditional cuisine, and dairy products from many parts of the world are rich sources of LAB due to their traditional manufacturing techniques, which frequently involve spontaneous fermentation (Sharma and Bajwa, 2021). LAB contribute to many foods’ distinctive nutritional and taste characteristics and their colonization of the digestive system, decreased blood cholesterol, competition with pathogens and enhanced antibody production positively benefit human health (Kaur et al., 2012). It inhibits the growth of bacteria that would otherwise compromise the food’s microbiological safety and reduce its shelf life. Numerous studies have reported that specific LAB species, including *Lactobacillus plantarum*, *L. paracasei*, and *L. fermentum*, possess the capacity to generate BS (Zahed et al., 2022). Among these, *L. plantarum*, which is Generally Regarded as Safe (GRAS), has shown the most promising results in terms of BS production (Aboelkhair et al., 2022). *L. plantarum* strains can survive in the gastrointestinal tract, attach to epithelial cells, and, most significantly, are safe for animal and human health (Lara et al., 2022a). The BS obtained from *L. plantarum* has shown potent anti-adhesive, antimicrobial, antifungal, and antiviral properties (Thakur et al., 2023). The antibacterial activity has been demonstrated against harmful pathogens like *Escherichia coli*, and *Staphylococcus aureus* (Kaur and Kaur, 2019; De Giani et al., 2021). Different studies involved in the production of BS-LAB have reported a lot of structural and composition diversity using techniques such as FTIR, NMR, GC–MS, LC–MS, TLC, etc. (Karnwal et al., 2023). The synthesis of BS by *L. plantarum* is notably influenced by multiple factors, including carbon source, pH levels, and temperature. *L. plantarum* produces a higher amount of BS in comparison to *L. fermentum* and *L. acidophilus* and also higher yield was obtained by using lactose as a carbon source (23.8%) than glucose (12.8%) within 48 h. under stationary conditions with highest production levels achieved during the exponential growth phase (Kumar et al., 2021). *L. plantarum 1625* biosurfactant has shown antibacterial efficiency against a variety of clinically relevant infections in addition to surface activity. The potential of biosurfactants to damage microbial cell membranes and increase their susceptibility to immune responses or antimicrobial drugs may be the cause of this antimicrobial activity (Nataraj et al., 2021). In addition, the BS derived from LAB have antibiofilm properties that make it noteworthy. Biofilms are intricate microbial communities that are protected from antibiotics by an extracellular polymeric matrix (Thi et al., 2020). They also play a role in the persistence of diseases. Biosurfactants provide a viable approach to inhibiting biofilm-associated illnesses and boost the effectiveness of traditional antimicrobial treatments by focusing on biofilm development and dispersal (Salman and Alimer, 2014). Furthermore, the issue of antibiotic resistance can be addressed with the use of LAB derived BS. The evolution of bacterial strains becoming resistant to synthetic antimicrobial drugs presents a serious risk to public health. BS produced by LAB, on the other hand, can act as alternate antibacterial agent with minimum risk of developing resistance (Alara and Alara, 2024).

There are significant benefits to the economy and ecology from LAB BS. In terms of cost, these biosurfactants provide a reasonably priced alternative to synthetic surfactants. They may be produced using fermentation processes and renewable resources, which are often less expensive and more ecologically friendly than synthetic surfactants produced from hydrocarbons. The pharmaceutical, cosmetic, and agricultural industries, which rely heavily on surfactants, stand to gain the most from this cost decrease. The capacity of LAB to create biosurfactants utilizing agricultural waste or by-products as substrates further reduces production costs and helps with waste management solutions (Kumari et al., 2023). The sustainable goals are aligned with their prospective applications in different industrial and environmental sectors, including biodegradability, and the potential to minimize antimicrobial resistance making them a viable choice (Nagtode et al., 2023).

The objective of this study is to describe that biosurfactant produced by *L. plantarum strain* 1625 shows antibacterial and antibiofilm properties and assess its possible uses in the treatment of microbial diseases. This work intends to aid in the development of an antimicrobial and antibiofilm agent that is capable of inhibiting various biofilms responsible for multidrug-resistant infections formed in the medical equipment.

## 2 Materials and methodology

### 2.1 Isolation and screening of isolates for BS production

LAB was isolated using MRS (de Mann Rogosa Sharpe) agar from several fermented foods and dairy products. For 48 h., the plates were incubated at 37°C under anaerobic conditions. The initial identification was carried out based on morphological and biochemical characteristics (Srinivash et al., 2023). The cultured LAB isolates were kept at 37°C for 24 h. in 50 mL of MRS broth to enhance the production of BS. To separate the cells, the bacterial culture was centrifuged at 10,000 *g* for 5 min. The resulting supernatant, obtained through a 0.2 μm filtration, was used to assess BS activity. To confirm the presence of BS, several screening methods were used, including oil displacement, drop collapse, and emulsification index (John et al., 2021). The oil displacement method involved adding oil to a petridish that was filled with water and then measuring the amount of oil displacement by adding a drop of cell-free supernatant (CFS). The size of the displaced area was measured three times to ensure precision. The negative control was prepared using an uninoculated broth. The surface tension of hydrophobic and aqueous surfaces is assessed in the drop collapse experiment by pipetting a 25 μL droplet of extracted BS onto parafilm and examining the droplet’s expansion and flattening for 1 min. 2 mL of engine oil and 2 mL of CFS were vortexed vigorously at 2000 rpm for 2 min to evaluate the emulsification index. The mixture was then left to remain undisturbed for a period of 48 h. The resulting emulsification index, represented as a percentage, was calculated using the specified formula, i.e.,


$$E48\;\left(\%\right)=\frac{\mathrm{Height}\;\mathrm{of}\;\mathrm{emulsion}\;}{\mathrm{Total}\;\mathrm{height}\;\mathrm{of}\;\mathrm{the}\;\mathrm{solution}}X100$$

### 2.2 Haemolytic assay

Isolated strains were screened on Sheep blood/agar plates incubated at 37°C for 48 h. Hemolytic activity was detected by the presence of a definite clear zone around a colony (Monika et al., 2017).

### 2.3 Probiotic attributes of bacterial isolates

The isolate exhibiting a greater capacity to produce BS was subjected to screening for certain probiotic traits, such as acid and bile tolerance, gelatinase test capability, and bacterial growth pattern. In the acid tolerance testing, LAB was inoculated into each tube, and the pH of the MRS broth was adjusted from 1.0 to 7.0. All tubes were then incubated at 37°C. For the bile tolerance assessment, varying concentrations of bile salts (0.1–0.3%), were introduced into the MRS broth, which was then inoculated with LAB. MRS broth devoid of bile salt was utilized as the control. To evaluate the proliferation of bacteria, the absorbance was measured at 600 nm using a spectrophotometer at 2 h. intervals in triplicate. For the gelatinase assay, the test tubes containing gelatin media were stab-inoculated with LAB. The tubes were incubated at 25°C for 1 week, and gelatin’s liquefaction was monitored daily. The tubes were submerged in an ice bath for 15–30 min to confirm the liquefaction caused by gelatinase activity, as gelatin typically liquefies at temperatures above 25°C. The tubes were then inverted to determine whether gelatin had been hydrolyzed. To track the growth of LAB, the isolate was added to MRS broth and incubated at 37°C. Every 2 h. The absorbance at 600 nm was measured to assess the growth (Hassanzadazar et al., 2012).

### 2.4 Identification of bacterial isolate using 16S rRNA sequencing

A potential isolate showing the highest BS production and probiotic attributes was molecularly identified using 16S rRNA gene sequencing. After obtaining the sequences, a BLASTN search was performed in the NCBI database to identify sequence homology with 16S rRNA gene. Mega X software was utilized in the construction of the phylogenetic tree (Balakrishnan et al., 2023).

### 2.5 Production and extraction of BS

To produce BS, a culture of LAB was incubated overnight and then added to 100 mL of MRS broth. This mixture was kept at a temperature of 37°C for 48 h. at 120 rpm. After the 48 h., the broth was subjected to centrifugation at 6500 *g* for 20 min. at a temperature of 4°C. This process resulted in the separation of the CFS. The ethyl acetate precipitation method was used to extract the biosurfactants. The pH of the cell-free supernatant (CFS) was adjusted to 2 with 1 N HCl, it was transferred to a separatory funnel and an equivalent amount of ethyl acetate was added. Three replications of phase separation were performed on the mixture by shaking. The organic phase was separated, treated with anhydrous sodium sulfate to remove water, and then concentrated at 35–40°C in a rotary evaporator to get the CB extract. The CB obtained was freeze-dried and the yield was recorded in mg mL^−1^ (Ghazi Faisal et al., 2023).

### 2.6 Characterization of BS

#### 2.6.1 Chemical characterization of BS

The Biuret test confirmed the existence of lipopeptides in the CB. The ammonium molybdate precipitation method was utilized to identify the presence of phospholipids. The presence of phospholipid BS was indicated by the emergence of a yellow color, which was followed by a gradual formation of a fine yellow precipitate. Amino acids were detected through the Ninhydrin test (Zargar et al., 2022). To check the hydrolysis of fats, the small quantity of CB was mixed with 2 mL of a 2% NaOH solution and was well agitated.

#### 2.6.2 Ionic characterization of BS

The BS ionic charge was assessed using the agar double diffusion technique. Two sets of wells were created using a 1% (w/v) agar solution. The CB was applied to one row of wells, and a reference material with a known ionic charge was added to the other row. BaCl_2_ was used as a cationic surfactant, while SDS was used as an anionic surfactant. During 48 h. of incubation at 37°C, the development of precipitation lines between the wells was observed (Nikolaeva et al., 2022).

#### 2.6.3 Structural characterization of BS

##### 2.6.3.1 FTIR analysis

The functional groups and their stretching were identified by FTIR spectroscopy using a Shimadzu 8,400 device (Japan). The spectrum had a resolution of 4 cm^−1^ and covered a range from 4,000 cm^−1^ to 650 cm^−1^ (Sabarinathan et al., 2021; Ahuja et al., 2022).

##### 2.6.3.2 GC–MS analysis

With the use of a VF-5MS column-equipped gas chromatograph-mass spectrometer, the composition of the BS sample was determined. The temperature of the column was initially dropped to 100°C for 1 min., and then it was gradually raised to 270°C at a rate of 30°C min^−1^, staying at this level for 10 min. The quadrupole, ion trap, and transfer line were set to 280°C, 230°C, and 160°C, respectively. An injection of 50 μL of BS occurred at 270°C at the inlet. Helium gas flowed at a rate of 1.2 mL min^−1^ (Vidur and Saharan, 2016).

### 2.7 Antimicrobial activity of isolated BS

The antibacterial efficacy of the CB against *Staphylococcus aureus* MTCC 10449, *Escherichia coli* MTCC587 and *Pseudomonas putida* MTCC-1655 was evaluated using the agar well diffusion assay. *E. coli, S. aureus*, and *P. putida* were cultured for 24 h at 37°C and 120 rpm. Then, 100 μL of each culture was added to the freshly prepared broth and incubated again until the absorbance reached 0.4 A.U. After that, the cultures were spread on Mueller Hinton (MH) agar plates. On the MH agar plates, 5 mm diameter wells were made. Different concentration of BS (0.1, 0.15, 0.175, 0.25 mg mL^−1^) were taken and then 50 μL of CB [dissolved in 50% ethanol (having no antibacterial properties)] was added to each well (Hassanzadazar et al., 2012). The inhibitory zone’s diameter was then determined and the percentage inhibition was using the formula (Liang et al., 2014).


$$\mathrm{Percentage}\;\mathrm{inhibition}=\frac{\left(1-\left(\mathrm{Control}-\mathrm{sample}\right)\right)\;}{\left(\mathrm{Control}\right)}X100$$

### 2.8 Antibiofilm activity of isolated BS

To check the antibiofilm activity of the BS two methods were used, i.e., microtiter plate assay and co-incubation assay with slight modifications.

- Microtiter plate assay

To initiate the experiment, a small quantity of *S. aureus* was introduced into 3–5 mL of nutrient broth, which was subsequently transferred to an incubator set at a temperature of 37°C for a duration of 24 h. 100 μL of distilled water was added to the first well, which was designated as the negative control. Subsequently, 50 μL of *S. aureus* and 50 μL of water designated as a positive control were introduced into the well. Next, 50 μL of LAB BS2 and 50 μL of *S. aureus* was added to the well. Following the mixture of 50 μL of BS extract (dissolved in ethanol) and 50 μL of *S. aureus*, the plate was sealed and allowed to incubate for a full day at 37°C. Cells were washed from each well three times with distilled water. Each well was then stained with 125 μL of 0.1% crystal violet, left for 10 min., and then cleaned with distilled water before being dried. Following the addition of 200 μL of ethanol to each well and mixing for 15 min, the optical density (OD) at 600 nm was determined (Sharma and Kaur, 2013).

- Co-incubation assays

The biofilm inhibition assay was performed using silicon tubes as described by Sharma and Saharan (2016), against three biofilm-forming strains *viz. E. coli* MTCC 1587, *S. aureus* MTCC 1049, and *P. putida* MTCC 1655. Overnight cultures (0.1 mL) of the three strains *E. coli, S. aureus,* and *P. putida* were inoculated into 3 test tubes containing freshly prepared Nutrient broth and 4 cm sterile silicon catheter tube and 1 mL of BS (0.25 mg mL^−1^). The other set of test tubes containing all components except BS served as the positive control for the biofilm formation, respectively. Both sets underwent a 24 h. incubation period at 37°C. The silicone tubes were taken out and cleaned with distilled water after this incubation. After air drying, the silicone tubes were stained for 20 min, using 0.1% crystal violet solution. The silicone tubes were washed twice with distilled water and air dried for 30 min, at room temperature. The color variations were appropriately documented and SEM images were taken for the further confirmation of biofilm inhibition.

### 2.9 Optimal conditions for BS production

The effects of temperature, pH, and different substrates on the production of BS were studied. 0.1 N HCl or 0.1 N NaOH were used to alter the pH values in MRS from 3 to 9. Temperature effects were evaluated by incubating the LAB-inoculated MRS medium at temperatures ranging from 15 to 60°C. To examine the effect on BS production using various biowaste, pineapple peels, orange peels, groundnut shells, sugarcane bagasse, and coconut husk, were used as a substrate. A 1% overnight culture of LAB was added to each flask as an inoculant. After 48 h. of incubation at 120 rpm, the emulsification activity was quantified (Mnif et al., 2023).

## 3 Optimization of BS production using response surface methodology

The Box bechken design (BBD) optimization method was utilized to examine the impact of various factors on the response. The three process-independent factors, namely the culture medium’s pH (A), incubation temperature (B), and orange peel concentration (C), were considered in this analysis. To analyze the data, a quadratic model was used. In the experiment, each parameter was examined at three different levels (−1, 0, +1), encompassing the lowest and highest extents 3 variables (Supplementary Table 1). 17 trials were designed, and a second-order polynomial equation was used to determine the responses in the form of emulsification index (48 h). A multiple-regression technique was then used to model the data. After that, tests were conducted using the suggested optimized values for every variable found by RSM to verify the actual emulsification index in comparison to the possible emulsification index. The results of the experimental design were subsequently put into action and analyzed using Stat-Ease’s Design-Expert^®^ version-8 software (Bellebcir et al., 2023).

## 4 Results and discussion

### 4.1 Isolation and screening of bacterial isolates for BS production

Twenty-six bacterial isolates were procured from a variety of sources, including dairy products, fermented foods, and the first milk from cows, called colostrum. Out of the initial 26 bacterial isolates, 8 were selected based on the biochemical and morphological tests for lactic acid bacteria. These 8 isolates were further screened for the production of biosurfactant. All 8 bacterial isolates showed Gram-positive status and negative catalase and oxidase test results which corresponds to LAB. Similarly, Ngene et al. (2019) additionally demonstrated that several isolates of LAB from fermented food in Nigeria showed negative reactions to catalase and oxidase assays. In the BS production screening tests out of all the 8 isolates, LAB-BS2 showed the best results for biosurfactant production and was chosen for further analysis and investigation as shown in Supplementary Table 2. LAB-BS2 created a separate zone with a diameter of 1.13 ± 0.29 in the oil displacement test and flattened the drop in the drop collapse test. Additionally, LAB-BS2 increased the emulsification index from 40 to 80.5% in 48 h, emulsifying the hydrocarbons. Ghasemi et al. (2019) observed that strain SHU1593 (*Pediococcus dextrinicus*) exhibits a lowering of the emulsification activity of hydrocarbon by producing lipoprotein a BS compound. Hence, LAB-BS2 could be used for additional research after demonstrating the greatest output of BS in screening tests.

### 4.2 Haemolysis assay

LAB-BS2 were ᵧ haemolytic (no haemolysis). So the bacteria was found to be non-pathogenic as no definite clear zones were observed around the colonies as shown in Supplementary Figure 1. Similar observations were made for strains of *L. paracasei subsp. paracasei*, *Lactobacillus spp*., and *L. casei* isolated from dairy products, which exhibited gamma hemolysis (Monika et al., 2017).

### 4.3 Probiotic attributes of isolated bacteria

When screened for different probiotic attributes, LAB-BS2 showed positive results. LAB-BS2 showed high tolerance in acidic conditions (pH 1 to 3) till 4 h but after 4 h, their viability was relatively low. After 4 h. of incubation, the bacteria at pH 4 to 5.5 displayed stagnant growth. Then a decreased bacterial count was detected compared to the control at pH 6.8. At pH 6.8 a slow increase was observed during the first 4–6 h. According to another investigation, *L. casei/paracasei* vitality was significantly reduced after being exposed to pH 2 simulated stomach juice for 90 min. Ruiz-Moyano et al. (2019) proved that the LAB can survive in an acidic environment but not more than 2–4 h. The bacteria were viable at lower pH for about 4 h and after that, their viability started decreasing. LAB BS2, also exhibited resistance and viability at varying concentrations of bile salt, as illustrated in Supplementary Figure 2. This is in line with a previous study where *L. plantarum* HLX37 can withstand elevated levels of bile salt (Guan et al., 2017). LAB-BS2 did not show liquefaction of the gelatin and thus was negative for gelatinase production.

The growth of the LAB-BS2 on MRS followed logarithmic growth in which the lag phase lasted from 0 to 2 h. log phase for 2–8 h. stationary phase from 10 to 14 h. and reached the declined phase after 14 h. as shown in Supplementary Figure 3. Similar pattern of growth curve for *L. plantarum* was obtained by Kim et al. (2023). The exponential growth phase of LAB lies between the second and eighth hr. at 37°C, and it continues up to the tenth hour (Smetanková et al., 2012).

### 4.4 Identification of LAB BS2

**Figure 1:**
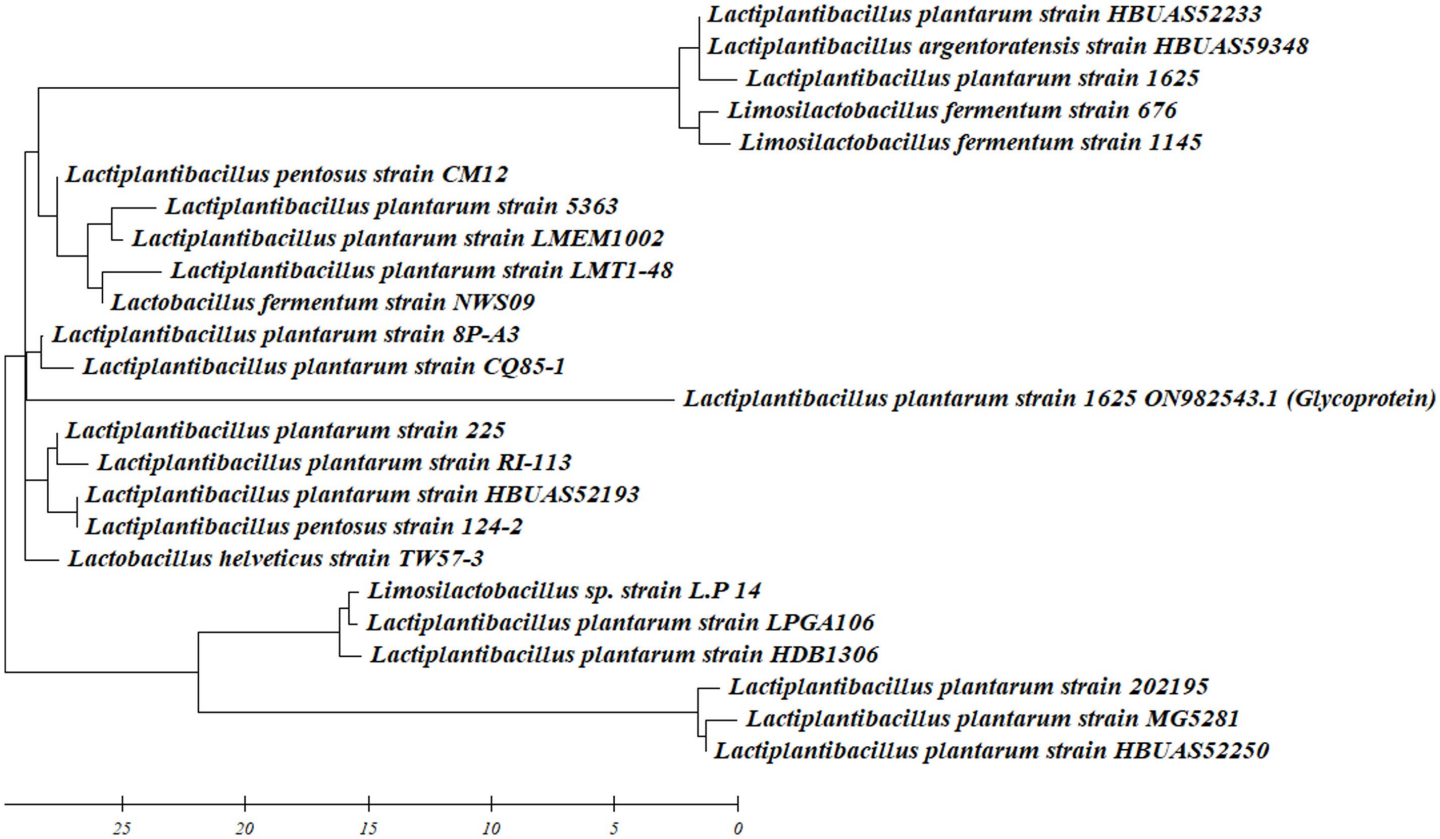
Phylogenetic tree of *L. plantarum* 1625 including retrieved sequence from Genbank data base.

The LAB BS2 showed higher production of BS and was also positive for different probiotic attributes. Therefore, through the implementation of 16S rRNA sequencing, LAB-BS2 was identified as *L. plantarum* 1625. The obtained information has been duly recorded and shared on Genbank under the accession number ON982543. Similar approach for LAB identification was demonstrated by Mouafo et al. (2023). The phylogenetic tree was constructed using Mega XI software is shown in Figure 1.

### 4.5 Production and extraction of BS

In this study, *L. plantarum* 1,625 was employed to produce BS using MRS broth, and similarly, earlier isolate BS from LAB. A CB was extracted using ethyl acetate precipitation and a yield of 1.24 mg mL^−1^ was obtained.

### 4.6 Characterization of BS (BS)

#### 4.6.1 Chemical characterization of BS

The BS was composed of proteins, lipids, and carbohydrates and the most plentiful constituents are carbohydrates, followed by proteins and lipids. They are usually produced as glycolipid, glycoprotein and glycolipoprotein. To confirm the existence of lipopeptides, phospholipids, and amino acids in the BS, a sequence of tests, including the biuret test, phosphate test, and ninhydrin test, were conducted. The colorless phosphate test solution became yellow in this experiment, indicating a positive phospholipid result. According to preliminary identification findings, BS used in the study may belong to the phospholipid class. To detect the existence of amino acids, a biuret test was executed. The formation of a purple-colored complex in the test tube signified the existence of amino acids within the sample. When the lipid in the BS is saponified by NaOH, it passes the lipid saponification test, proving that lipids are present in the isolated BS. The characterization tests showed that isolated BS contains fatty acids, lipopeptides, phospholipids, and amino acid. Similar results were observed during the characterization of biosurfactants by Zargar et al. (2022) using TLC plates loaded with BS. Their findings verified the presence of fats and carbohydrates.

#### 4.6.2 Ionic characterization of BS

##### 4.6.2.1 Agar double diffusion method

The precipitation lines were apparent with cationic BaCl_2_ and on the other hand, no precipitation lines were observed with SDS (anionic substance) in agar double diffusion assay, which indicated that BS is anionic in nature.

#### 4.6.3 Structural characterization of BS

##### 4.6.3.1 FTIR analysis

**Figure 2:**
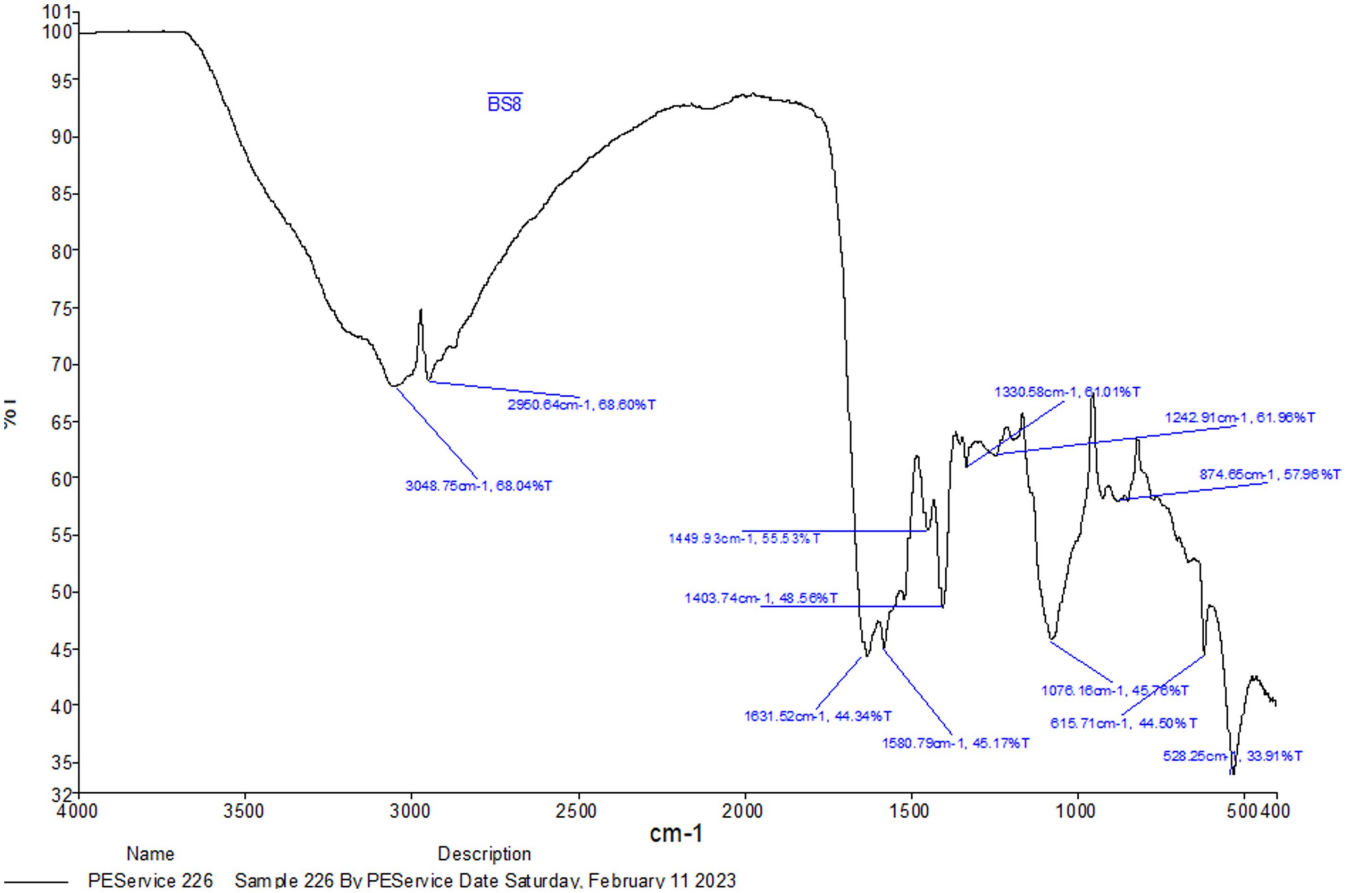
FTIR analysis of extracted biosurfactant from *L. plantarum* 1625.

The peaks and their corresponding chemical groups linked to the BS obtained by FTIR are shown in Figure 2. The = C-H stretching, C-H asymmetric stretching of CH_3_, amide I bond (C=O stretching), CH_3_ bending, and phosphate were all reported to have strong peaks at 3,048 cm^−1^, 2,950 cm^−1^, 1,631 cm^−1^, 1,580 cm^−1^, 1,449 cm^−1^, 1,403 cm^−1^, 1,242 cm^−1^, and 1,076 cm^−1^, as shown in Supplementary Table 3. The presence of protein amide moieties was demonstrated by the peak at 1075 cm^−1^. Proteins’ NH group, C=O bond stretching, and NH bond bending are represented by the three main peaks in the spectra. These bands are observed in the wavelength ranges of 1,500–1,620 cm^−1^, 1,500–3,600 cm^−1^, and 3,000–3,600 cm^−1^, respectively, and indicating the presence of proteins in the samples. In the FTIR spectra of glycolipoproteins BS, occasional absorption in the 1,500–1,620 cm^−1^ range is noticed. Furthermore, the peaks observed at 2,927, 2,929, and 2,924 cm^−1^in the FTIR spectrum correspond to C-H bands associated with CH_2_-CH_3_ stretching. There were notable bands at 3,124, 1,689, 1,400, 1,076, and 966 cm^−1^ in the N-4 biosurfactant’s FTIR spectra. The significant absorption peak at 1689 cm^−1^ is due to amino acid amine group stretching vibrations, or C=O vibrations. The peptide functional group’s stretching vibrations are responsible for the absorption peak at 3,124 cm^−1^. The compound’s complex structure is highlighted by the existence of conjugation between the carboxylic group and the amine groups of the amino acids, as shown by peaks at 1,076 and 1,689 cm^−3^ (Arora et al., 2019), which were found to be similar to the peaks obtained in ours research.

##### 4.6.3.2 GC–MS analysis

The GC–MS method was utilized to examine the molecular composition of the BS obtained from *L. plantarum* 1625. The obtained spectral peaks were then compared to information available in a reference library. The glycolipoprotein obtained from *L. plantarum* 1625 was primarily composed of long-chain fatty acids, with small amounts of polysaccharide fractions and protein. The most common fatty acid was found to be hexadecanoic acid. Other fatty acids were found to be octadecadienoic acid, methyl ester, methyl stearate and cyclononasiloxane.

Based on the observations the BS was found to be a glycolipoprotein. The distinct strains of *L. plantarum* produce distinct BS as a consequence of a combination of genetic, environmental, and evolutionary variables. These conditions may result in BS production diversity across various strains of the same species (Mouafo et al., 2018). In various studies, *L. plantarum* has been reported to produce different types of BS with different chemical natures such as polypeptides, fatty acids, glycolipids, glycoproteins and exopolysaccharides as shown in Supplementary Figure 4. Vallejo et al. (2021) reported the highest production of BS from *L. plantarum* as compared to other *Lactobacillus* sp. and *L. acidophilus* under stationary conditions after 48 h. There is great diversity in the chemical nature of the BS obtained from *Lactobacillus* sp.

Various strains of *L. plantarum* produce various BS, as seen in Supplementary Figure 5, which might be attributed to a variety of genetic and metabolic variables. Tchakouani et al. (2023), found that *L. plantarum* S5 produce BS in the form of polysaccharides, proteins, and phosphates which can play a crucial role in antibacterial activity against various food pathogens which are responsible for food spoilage. Abo Saif and Sakr (2020), characterized exopolysaccharides produced by *L. plantarum* 47FHE and studied their distinct properties like antibacterial, antiadhesive etc. Surlactin, exopolysaccharides, glycolipopetide and glycoprotein were produced by strains of *L. plantarum* which include *L. plantarum* 299v*, L. plantarum* 200,661, and *L. plantarum* CFR2194, respectively. Al-Seraih et al. (2022), used glycolipopeptide produced by *L. plantarum* SN35N as a bioemulsifier used in the food industries (Rodrigues et al., 2006; Merghni et al., 2017; Lim et al., 2020; Lara et al., 2022b).

Similarly, BS like rhamnolipids, surlactin and fatty acids produced by *L. plantarum* NCIMB8826, *L. plantarum* 27,172 and *L. plantarum* MBP001 were used as an antibacterial and antibiofilm agent against pathogens like *Pseudomonas aeruginosa, Chromobacterium violaceum* and *Staphylococcus aureus* which are responsible for causing life-threatening diseases (Yan et al., 2019; Hashim et al., 2022; Patel et al., 2022).

Bakhshi et al. (2018) and Madhu and Prapulla (2014), isolated and characterized glycoprotein, proteins and polysaccharide BS from *L. plantarum* PTCC 1896 and *L. plantarum* MCC 2156 and found their application as bioemulsifier.

#### 4.6.4 Antimicrobial and antibiofilm activities of BS

The CB was capable of inhibiting pathogens such as *S. aureus*, *E. coli* and *P. putida.* The BS formed the best inhibition zone at the concentration of 0.25 mg mL^−1^ against *S. aureus* which was 20 ± 0.03, *E. coli* was 11 ± 0.01 and *P. putida* was 14 ± 0.03 as shown in Supplementary Figure 6. Similarly, Sanam et al. (2022), indicated that the average inhibition zone of *E. coli* was 24.9 mm after 48 h. The percentage inhibition by BS at the highest and lowest concentrations was also studied. The results indicated a concentration-dependent inhibition effect for all the pathogens. In comparison to the antibiotic, which demonstrated 73.11% inhibition at 0.1 mg/mL and 99.44% at 0.25 mg/mL, the BS demonstrated 90.62% inhibition at 0.1 mg/mL and 98.7% at 0.25 mg/mL for *E. coli*. The BS showed 99.65% inhibition at 0.25 mg/mL and 97.88% inhibition at 0.1 mg/mL in *S. aureus*. At 0.1 mg/mL and 0.25 mg/mL, the antibiotic showed 94.35 and 98.32% inhibition, respectively. In comparison to the antibiotic, which showed 86.38% inhibition at 0.1 mg/mL and 97.49% at 0.25 mg/mL, the BS showed 94.22% inhibition at 0.1 mg/mL and 98.92% at 0.25 mg/mL for *P. putida*. These findings proved the great efficacy of the antibiotic and the BS in preventing the growth of *S. aureus*, *E. coli,* and *P. putida* with the BS often exhibiting more inhibition at the higher dose (0.25 mg/mL). This suggests that as concentrations of the antibiotic and BS rise, so does their efficacy. Therefore, although the BS shows promising activity as an antibacterial agent, its effectiveness increases with concentration. So the MIC of the BS was found to be 0.1 mg/mL. Similarly, the study conducted by Liang et al. (2014) demonstrates that the BS exhibits significant antimicrobial activity against *E. coli*, and *S. aureus*. It had exceptionally strong antibacterial activity at low concentrations and continued to do so even after being exposed to extremes in pH and temperature. This suggests that the biosurfactant TKU029 is a promising option for application as a new antibacterial agent in the treatment of bacterial illnesses.

The pattern that was observed during the antimicrobial activity was, as the concentration increased there was an increase in the zone of inhibition but after the concentration of 0.25 mg mL^−1^ no significant increase was observed. This mechanism was defined by Ndlovu et al. (2017), which is called the biphasic effect in which the effectiveness of biosurfactant starts decreasing beyond a certain concentration. This may be because at higher concentrations, BS starts accumulating and forming micelles, so the BS cannot interact with the targeted bacteria’s cell wall, leading to a decrease in the diameter of the inhibition zone. BS emulsify substances that are hydrophobic, which weakens the membranes and cell walls of microbes. Through surface tension reduction and surface modification, biosurfactants prevent the development of biofilms. Additionally, by dissolving the extracellular matrix, they can penetrate and disturb pre-existing biofilms. Additionally, biosurfactants can cause oxidative stress by producing reactive oxygen species (ROS) that harm cellular components and interfere with quorum sensing, which interferes with bacterial communication and the coordination of virulence factors. Because of these complex processes, BS produced by LAB are useful natural substitutes for synthetic antimicrobial agents, and they may also be able to lessen antibiotic resistance and combat infections caused by bacteria (Dini et al., 2024).

The antibiofilm activity of BS produced by *L. plantarum* was assessed using a microtiter plate assay against bacteria known for biofilm formation, which included *S. aureus, E. coli,* and *P. putida.* When studying for *S. aureus* biofilm inhibition, maximum absorbance was observed in the sample containing *S. aureus* and distilled water. The absorbance was lower in the case of *L. plantarum* 1625 supernatant mixed with *S. aureus.* When compared to this absorbance with the sample containing BS and *S. aureus,* it has a much lower absorbance.

**Figure 3:**
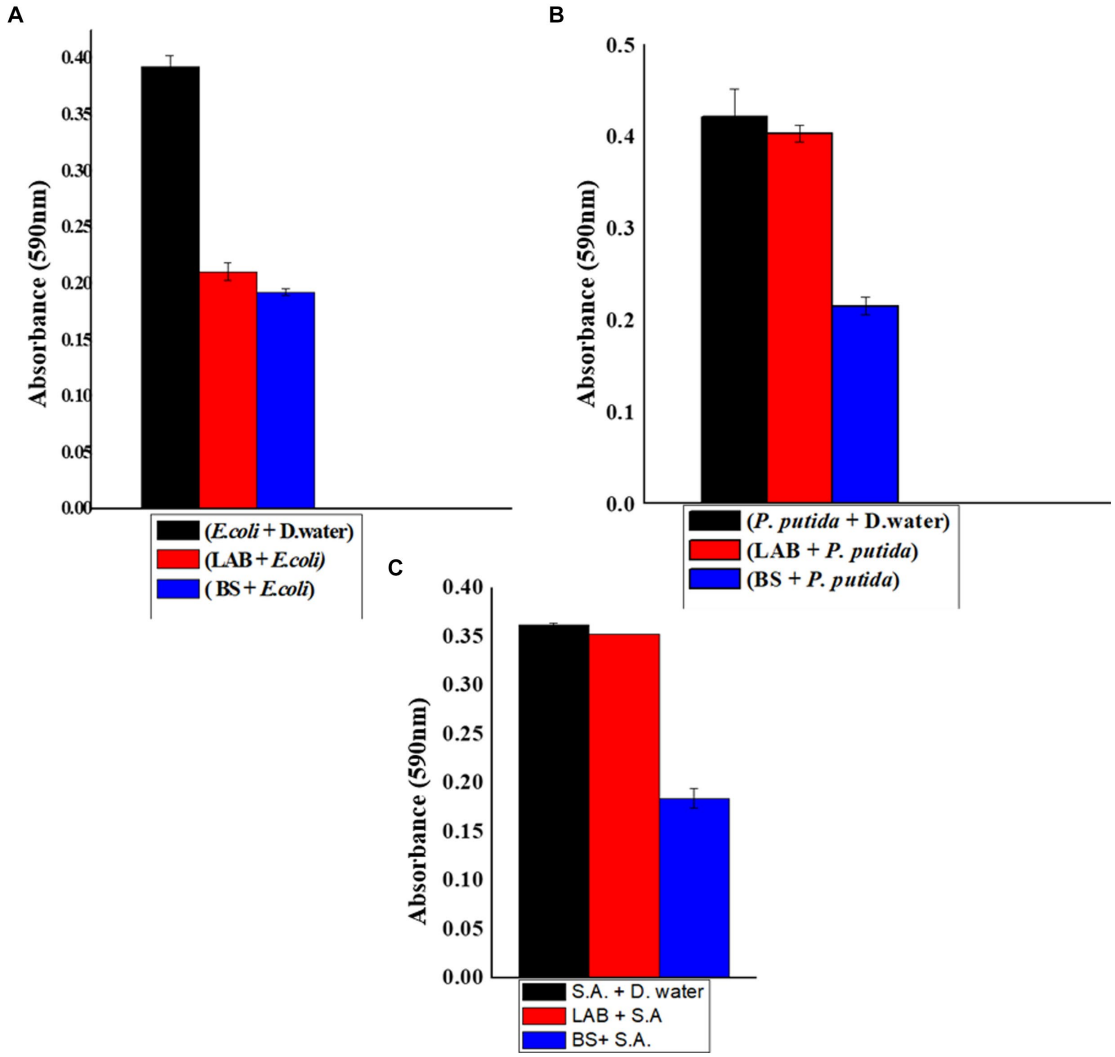
**(A)** Biofilm inhibition of biosurfactant against *E. coli* (1-*E. coli* + Distilled water, 2-*E. coli* + LAB, 3-biosurfactant + *E. coli*). **(B)** Biofilm inhibition of biosurfactant against *P. putida* (1-*P. putida* + Distilled water, 2-*P. putida* + LAB, 3-biosurfactant +*P. putida*). **(C)** Biofilm inhibition of biosurfactant against *S. aureus* (1-*S. aureus* + Distilled water, 2-*S. aureus* + LAB, 3-biosurfactant + *S. aureus*).

The results indicated that the treated samples exhibited decreased biofilm development and a reduced number of viable cells compared to the control a shown in Figure 3. The reduction in biofilm formation can be attributed to the decreased intensity resulting from a decline in the population of viable cells on surfaces treated with BS. This indicated that BS is capable of inhibiting the biofilm formed by *S. aureus* and *P. putida*. They cause membrane disruption, which increases membrane permeability, causes cell lysis, causes metabolite loss, and ultimately results in the death of the bacteria (Gurunathan et al., 2022).

The BS produced by *L. plantarum* could act as an antibiofilm agent against *P. putida, L. plantarum* can also inhibit the biofilm formed by *E. coli*. Comparable studies have demonstrated *L. plantarum* 1625’s ability to function as an antibiofilm agent against the pathogens that cause a variety of fatal illnesses (Arrioja-Bretón et al., 2020).

#### 4.6.5 Biofilm formation on silicon tubes (co-incubation assays)

**Figure 4:**
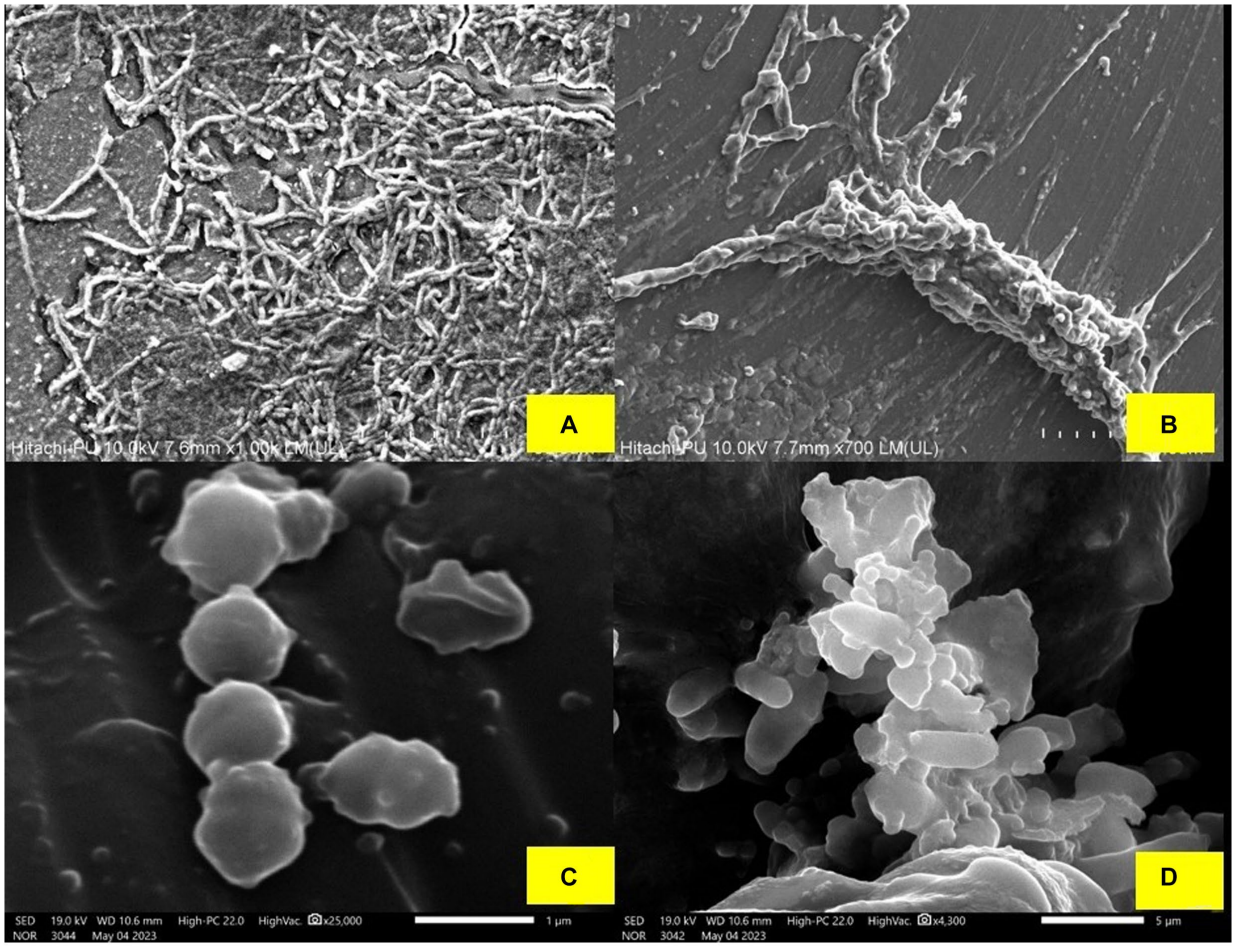
**(A)** Biofilm formed by the *P. putida*. **(B)** Minor destruction of *P. putida* biofilm after treatment with BS, **(C)** Biofilm formed by *S. aureus*, and **(D)** Minor destruction of *S. aureus* biofilm after treatment with BS.

In order to assess the formation of biofilms on medical-grade silicon tubes, biofilm-forming pathogenic strains were cultured on 4 cm sections of the silicon tubes that had previously been treated with BS produced from *L. plantarum* 1625. The silicon tubes were incubated with *E. coli, S. aureus* and *P. putida* which served as the positive control. By employing BS, the anti-biofilm characteristic was seen against the pathogens. When silicon tubes containing *E. coli, S. aureus*, and *P. putida* were stained with crystal violet for 20 min., biofilm formation was visible in the tubes. However, when the pathogens and BS were added at a concentration of 0.25 mg mL^−1^, biofilm formation decreased. The color difference between the two sets of silicon tubes as shown in Supplementary Figure 7, indicates that *L. plantarum 1,625 derived BS* inhibits the formation of biofilm and can be used as a precoating material in pharmaceutical industries. In the study conducted by Sharma and Saharan (2016) the biofilm inhibition by BS derived from *L. helveticus* was studied against pathogens responsible for causing various infections which included *E. coli, P. aeruginosa, S. aureus* and *C. albicans*. It was found that incubating BS with the pathogens reduced the biofilm formation as indicated by SEM images micrographs, captured with a resolution of 10.6 nm in Figure 4. The integration of BS into surface-cleaning methodologies presents a contemporary approach to maintaining the cleanliness of highly intricate laboratory and biomedical surfaces. This study is a step forward in developing new tactics to stop microbes from colonizing surgical instruments and silicone rubber prostheses.

#### 4.6.6 Optimal conditions for BS production

**Figure 5:**
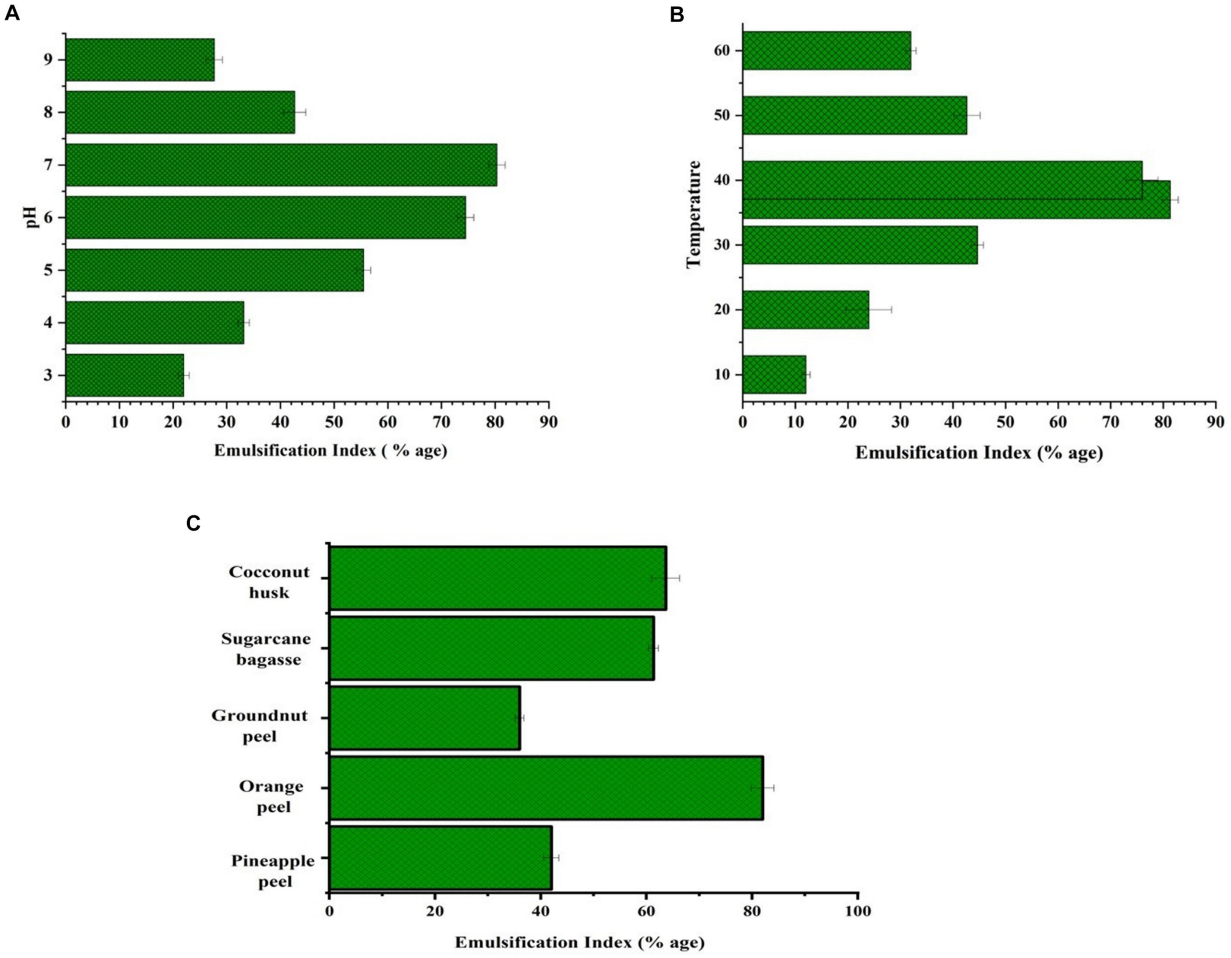
**(A)** Emulsification activity at different pH, **(B)** at different temperature **(C)** by using distinct biowaste.

As can be seen in Figure 5A, the ideal pH was discovered to be 7 with an emulsification index of 80.33% while optimizing the circumstances for the manufacture of BS. The BS emulsification activity is shown in Figure 5B for the following temperature ranges: 10°C, 20°C, 30°C, 37°C, 40°C, 50°C, and 60°C. The optimum temperature for the bacteria to produce BS was 37°C. The highest BS production by *L. helveticus* and *L. plantarum* has also been reported at 37°C (Zhou et al., 2015). Various biowaste such as coconut husk, pineapple peel, orange peel, groundnut shell, and sugarcane bagasse were tested for BS production. According to the findings, orange peel showed the highest BS production by giving emulsification activity, at 82% (Figure 5C).

Using *P. aeruginosa*, rhamnolipids were produced from a variety of agricultural waste products, such as banana waste, orange peelings, carrot waste, lime peelings, and coconut oil cake. According to George and Jayachandran (2013) orange peel was the most productive substrate, yielding 9.2 gL^−1^ of rhamnolipids. Likewise, *B. licheniformis* KC710973’s capacity to synthesize lipopeptides from potato, orange, and banana peels as well as two commercial extracts citrus peel being one of them was demonstrated. They discovered that using 4% orange peel resulted in 1.8 g/L of lipopeptides, making orange peel a cost-effective carbon source for enhancing lipopeptide production (Kumar et al., 2016).

#### 4.6.7 Optimization of BS production using response surface methodology

Three key variables were adjusted using the statistical model known as the Box–Behnken design (BBD) to improve the output of BS (Jayalatha et al., 2023): (A) pH, (B) temperature, and (C) the concentration of orange peel. According to the results, responsive emulsification activity (%) was achieved as shown in Supplementary Figure 8. Supplementary Table 4 shows the Box–Behnken Design matrix and its accompanying findings.

Parameter optimization was carried out using the BBD, which included three central points. The second-order polynomial equation was established as follows:


$$\begin{array}{l} E48\%\;\left(Y\right)=+64.2+1.75A-5.50\;B+3.75-2\mathrm{AB}\\ -5AC-0.5BC+10.85A^{2}-34.35B^{2}-6.85C^{2} \end{array}$$

Within the equation, Y denoted the response, specifically the emulsification index presented as a percentage. The variables A, B, and C were codified terms signifying the three tested variables: pH, temperature, and orange peel concentration, respectively.

When the linear terms were considered, three processed variables, pH (A) and temperature (B) showed the maximum influence on the emulsification index as compared to orange peel concentration (C). Upon considering the squared terms, it became evident that all the variables had a positive influence on the emulsification index. Additionally, the interactive effects of the variables AB (pH and temperature), BC (pH and temperature), and AC (pH and orange concentration) had a relatively minor positive impact on the emulsification index (Bayuo et al., 2020).

When compared to random error, the “Lack of Fit *F*-value” of 0.79 indicates that the Lack of Fit is not statistically significant. A model that fits the data well is indicated when the Lack of Fit is not substantial. The determination coefficient (*R*^2^), as indicated in Table 1, was 0.990, confirming a robust alignment between the model and the experimental data, denoting a strong fit.

**Table 1:** ANOVA table for RSM.

Source	Sum of squares	df	Mean square	*F* value	*p* value P > F	
Model	6511.73	9	723.52	84.69	<0.0001	significant
Ph	24.5	1	24.5	2.87	0.1342	
Temperature	242	1	242	28.33	0.0011	
Orange concentration	112.5	1	112.5	13.17	0.0084	
AB	16	1	16	1.87	0.2134	
AB	100	1	100	11.71	0.0111	
BC	1	1	1	0.1171	0.7423	
A^2^	495.67	1	495.67	58.02	0.0001	
B^2^	4968.09	1	4968.09	581.55	<0.0001	
C^2^	197.56	1	197.57	23.13	0.0019	
Residual	59.8	7	8.54			
Lack of fit	47	3	15.67	4.90	0.0795	not significant
Pure error	12.8	4	3.2			
Cor total	6571.52	16				

This result confirms that the model can account for 98% of the observed fluctuations in the response, leaving only 2% of variability unaccounted for over the experimental runs. The adjusted determination coefficient, with a value of 0.9792, further underscores the model’s strong statistical significance. The coefficient of variation, at 7.36%, highlights the model’s consistency. As shown in Supplementary Table 5, it is important to note that the “Pred R-Squared” value of 0.8825 closely resembles the “Adj R-Squared” value of 0.9792.

## 5 Conclusion

In conclusion, this study focused on the antimicrobial and antibiofilm properties of BS produced by LAB isolated from diverse fermented and dairy products. The BS that was produced has significant antibacterial and antibiofilm capabilities against *S. aureus, E. coli,* and *P. putida*, which indicate that it can be used in a variety of sectors such as biotechnology, and medicine. This study represents a forward-thinking advancement in discovering innovative methods to deter microbial colonization on surgical equipment and silicone rubber prostheses. The strain LAB BS2, derived from colostrum milk of cows, exhibited the highest production of BS which was confirmed using oil displacement, drop collapse and emulsification index. The isolated LAB BS2 exhibited various probiotic attributes viz. acid tolerance, bile tolerance, and gelatinase assay, proving its potential in various fields and was identified as *L. plantarum* 1625 using 16SrRNA gene sequencing. Through characterization techniques, the BS produced by *L. plantarum* 1625 was identified as an anionic glycolipoprotein. The structural composition of the BS was elucidated using Fourier Transform Infrared Spectroscopy (FTIR) and Gas Chromatography–Mass Spectrometry (GC–MS) analysis. This analysis revealed the presence of hexadecanoic acid and octadecadienoic acid chains. Moreover, this BS demonstrated excellent emulsification and antimicrobial activities. The BS production by *L. plantarum* 1625 was affected by varying pH, temperature, and substrate used during the production. The results showed that pH 7 and 37°C were the best conditions for BS production, with orange peel proving to be the most effective biowaste. The BBD and response surface methods produced a stable model with a substantial *R*^2^ value of 0.99, showing a good relationship between the experimental data and the model. Studying the various factors that influence BS production, especially biowaste can help to identify optimum conditions for high-yield production of BS. The BS obtained from *L. plantarum* 1625 demonstrated a diverse range of valuable properties, opening up new possibilities for their application in various industrial, environmental, and biotechnological contexts. This includes their potential use as biocontrol agents against pathogenic microorganisms. This study contributes to the understanding of LAB-derived BS and provides a foundation for their practical application in various pharmaceutical industries, however, more research is needed to determine its stability. Future research can concentrate on evaluating the BS stability in a range of storage and environmental conditions.

## Data Availability

The datasets presented in this study can be found in online repositories. The names of the repository/repositories and accession number(s) can be found in the article/Supplementary material.
