# Supplementary material for: Unveiling the antimicrobial and antibiofilm potential of biosurfactant produced by newly isolated Lactiplantibacillus plantarum strain 1625
Source: Front Microbiol. 2024 Sep 10;15:1459388. doi: 10.3389/fmicb.2024.1459388 (PMC11420119; doi:10.3389/fmicb.2024.1459388)
Supplement: Supplementary file 1 [file Table_1.DOCX]

**
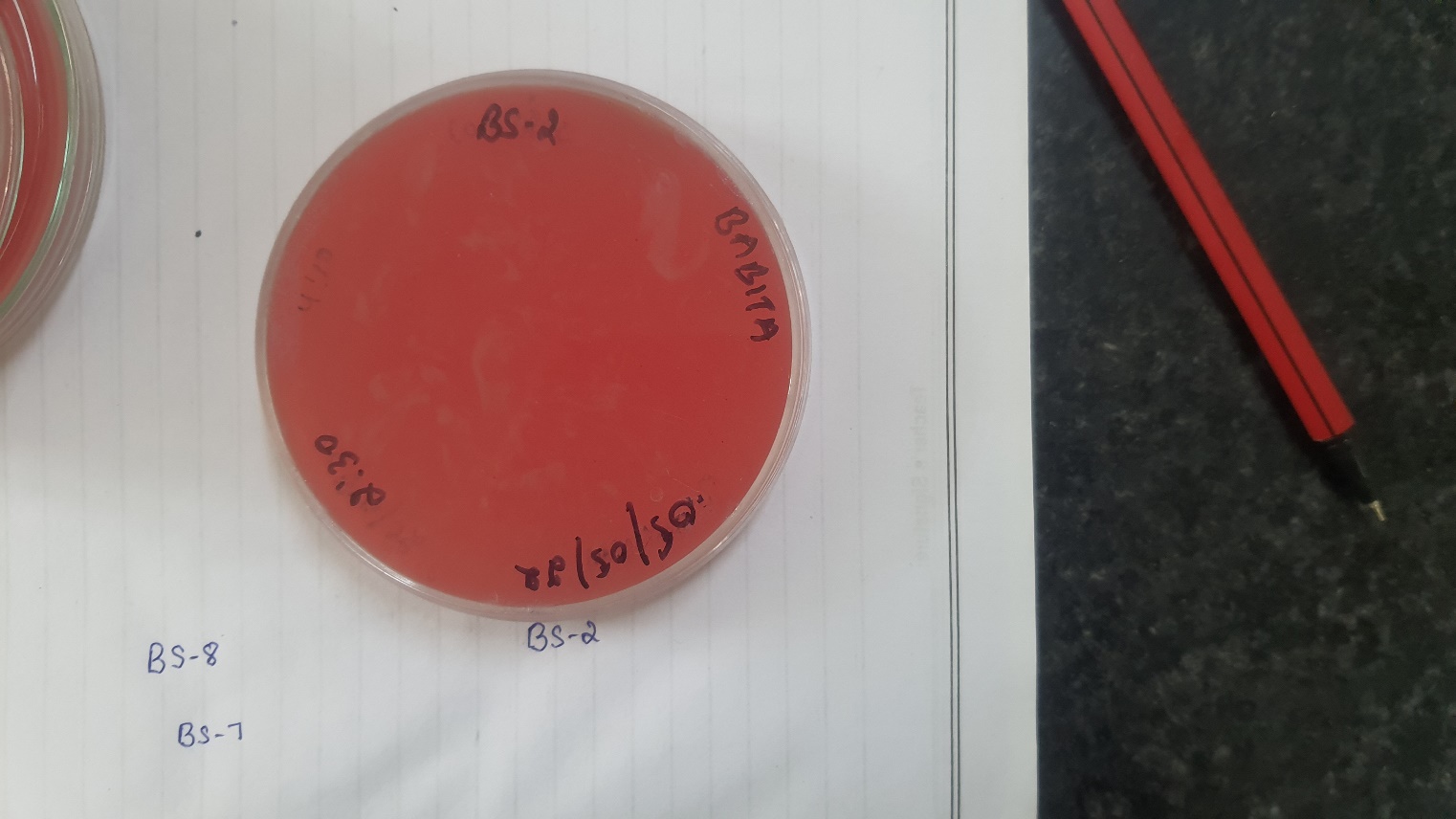
**

**Supplementary Figure 1.** Haemolysis assay showing negative result as no zone of clearance was observed

**Supplementary figure 2.** Survival of bacteria at different bile salt concentrations

**
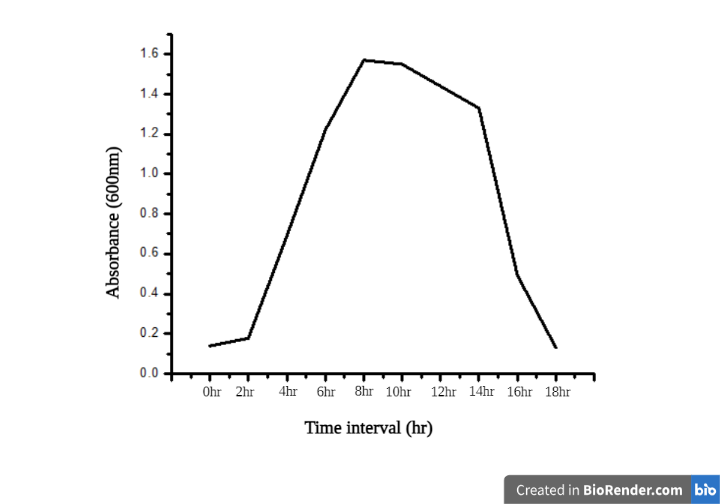
**

**Supplementary figure 3.** The growth phase of LAB BS2 bacteria


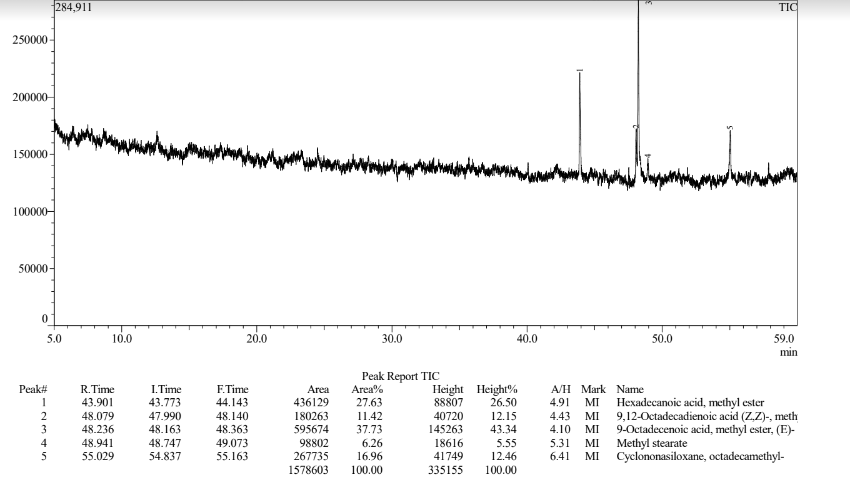


**Supplementary figure 4.** Peak analysis of GCMS

***
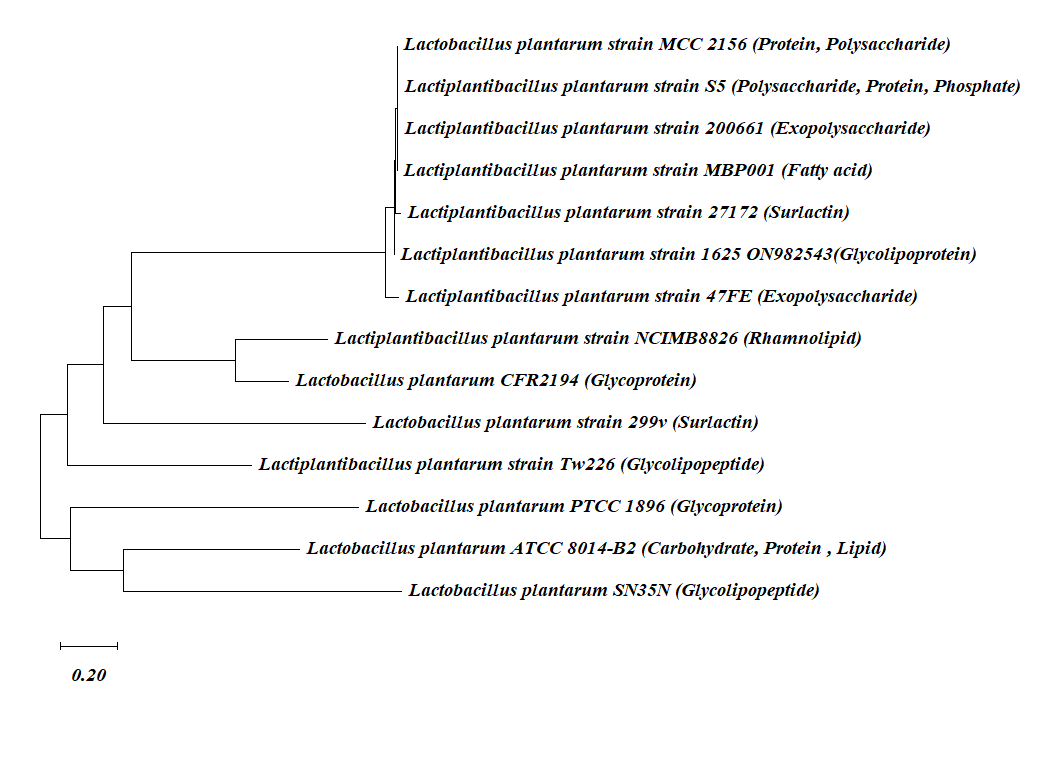
***

**Supplementary figure 5.** Distinct strains of *L. plantarum* showing variation in BS production

**c)**

**b)**

**a)**


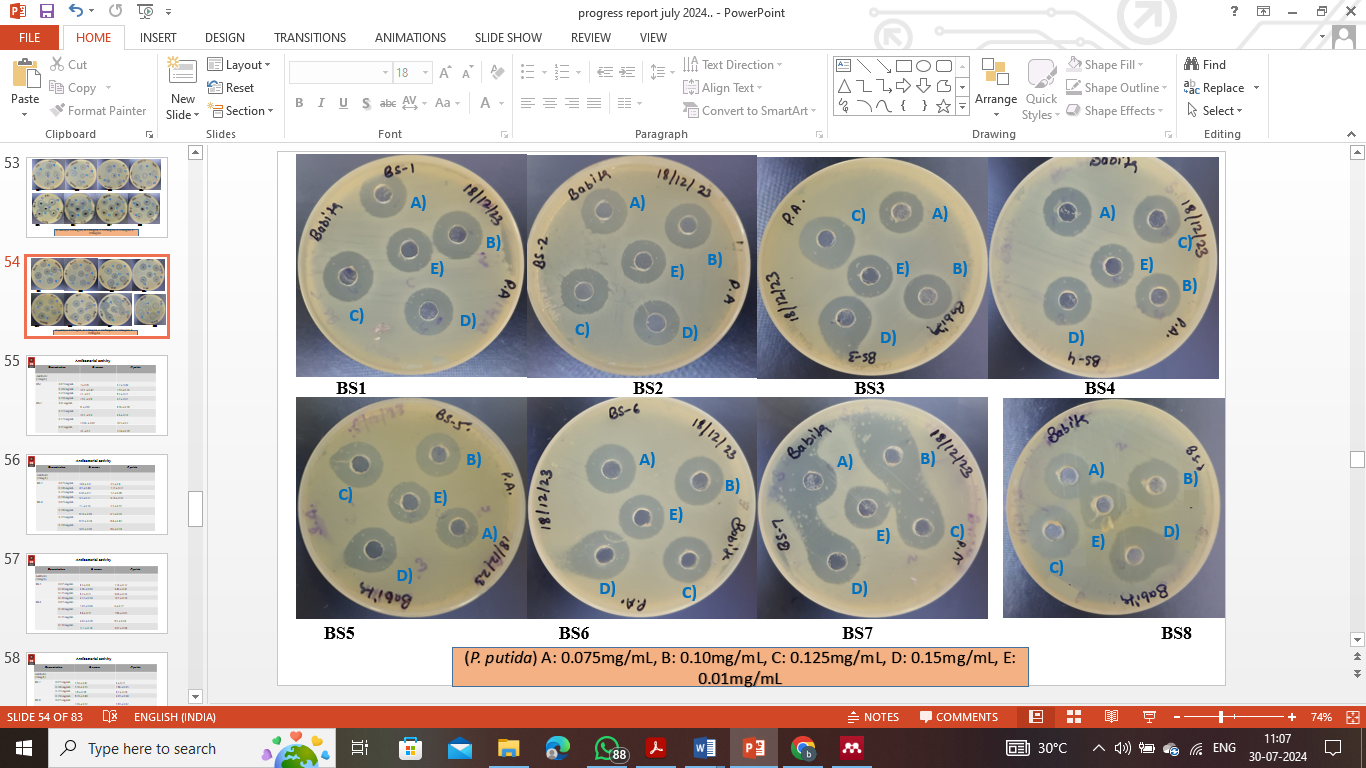

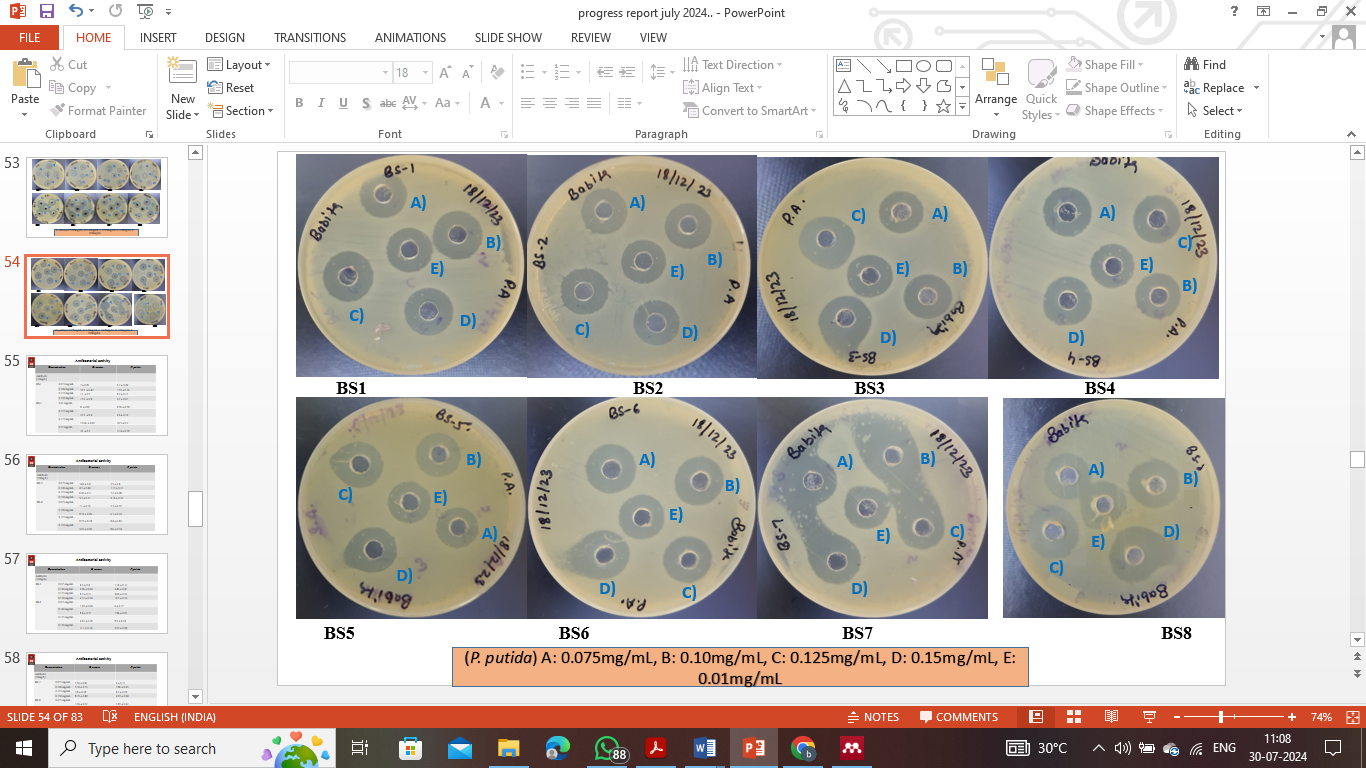

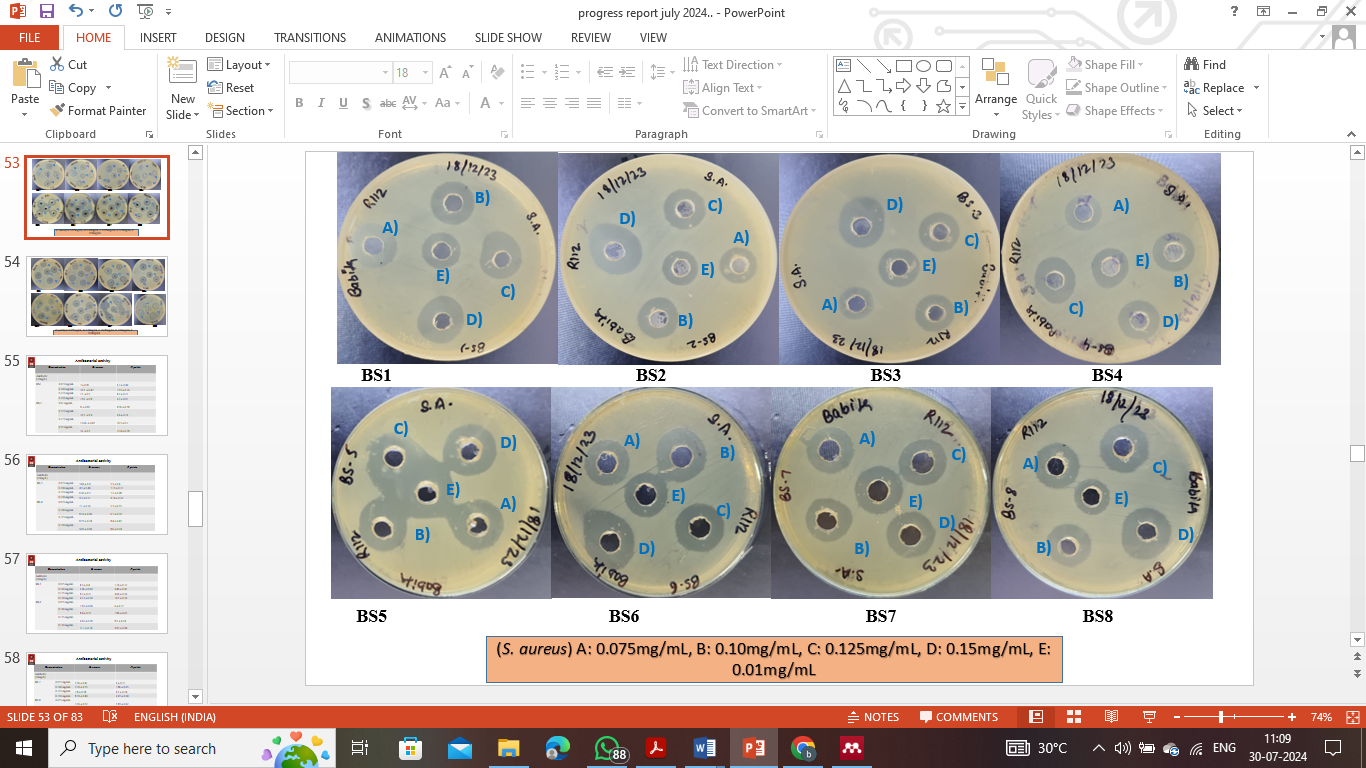


**Supplementary figure 6.** Agar well diffusion method for studying the antibacterial activity of BS at different concentration (A- 0.10, B-0.125, 0.175, 0.25 mgmL^-1^, E- 0.01 mgmL^-1^) azithromycin was taken as positive control against a) *S. aureus* b) *P. putida* and c) *E. coli*


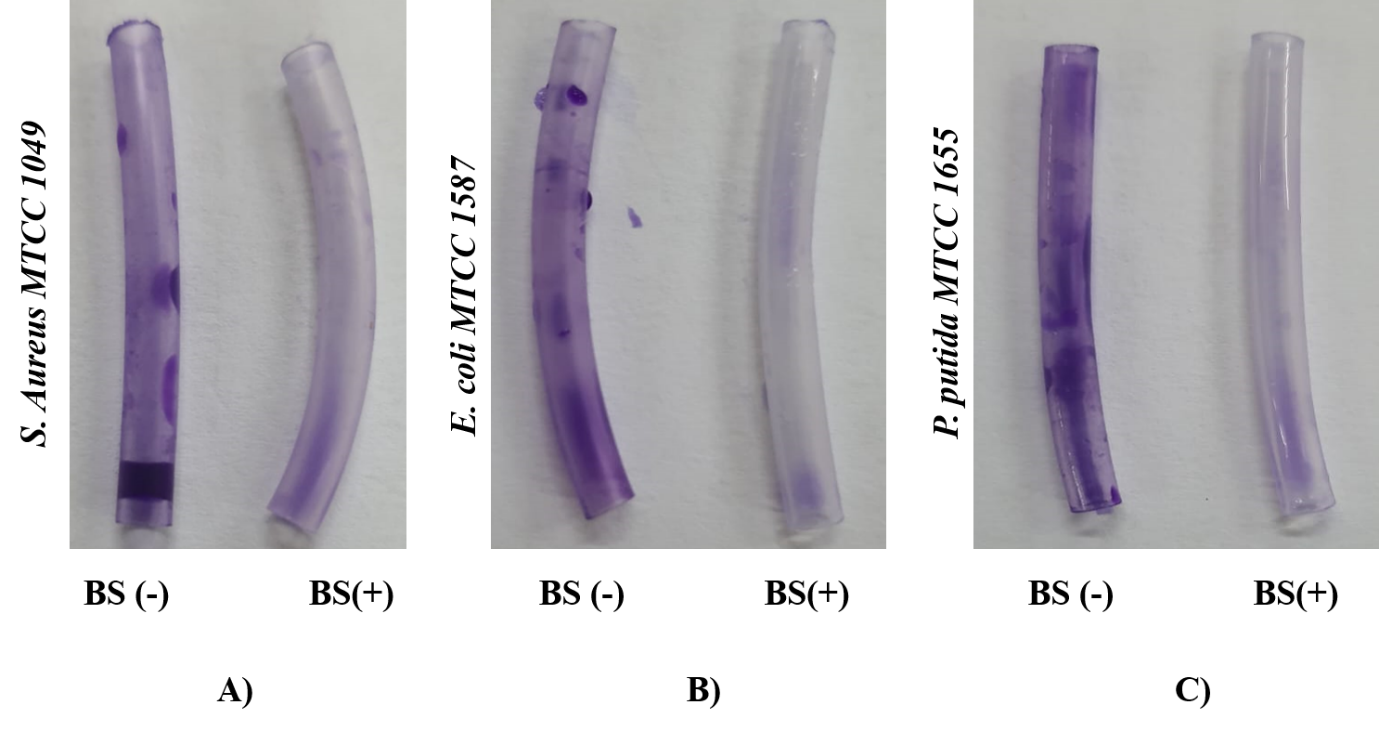


**Supplementary figure 7.** A) Biofilm formation by *S. aureus* in the presence and absence of BS derived from *L. plantarum* 1625, B) Biofilm formation by *E. coli* in presence and absence of BS derived from *L. plantarum* 1625, C) Biofilm formation by *P. putida* in the presence and absence of BS derived *from L. plantarum* 1625


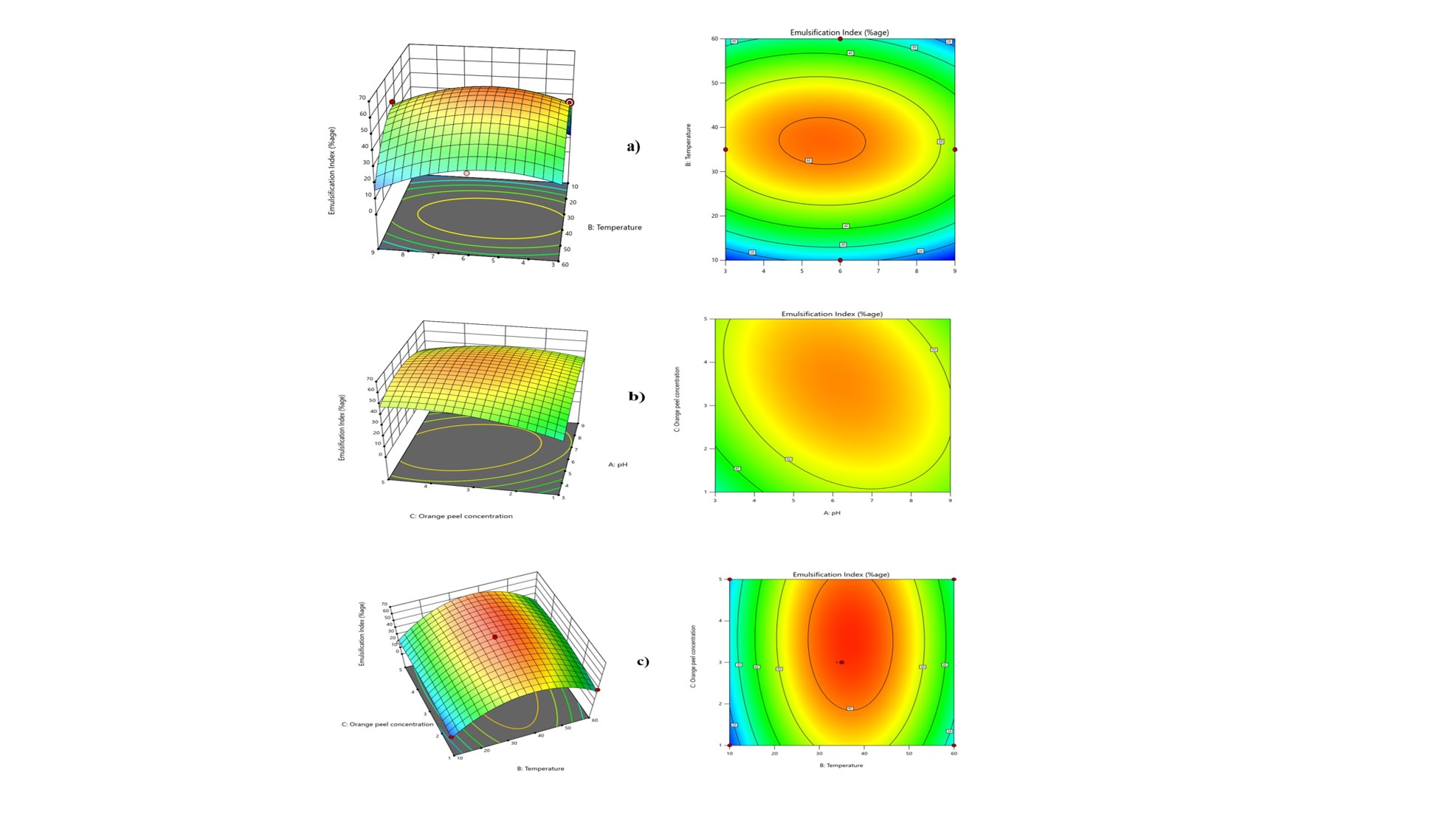


**Supplementary figure 8.** 3D response surface methodology (RSM) graphs and 2D contour plots showing the interaction of three factors a) AB (pH and temperature), b) AC (pH and orange peel concentration) and c) BC (temperature and pH)

**Supplementary table 1.** Box Behnken design for emulsification index

| **Std.** | **Run** | **Factor 1**  **A: pH** | **Factor 2**  **B: Temperature**  **(°C)** | **Factor 3**  **C: Orange peel concentration**  **(%)** |
| --- | --- | --- | --- | --- |
| 10 | 1 | 6 | 60 | 1 |
| 8 | 2 | 9 | 35 | 5 |
| 17 | 3 | 6 | 35 | 3 |
| 13 | 4 | 6 | 35 | 3 |
| 3 | 5 | 3 | 60 | 3 |
| 4 | 6 | 9 | 60 | 3 |
| 1 | 7 | 3 | 10 | 3 |
| 6 | 8 | 9 | 35 | 1 |
| 12 | 9 | 6 | 60 | 5 |
| 15 | 10 | 6 | 35 | 3 |
| 7 | 11 | 3 | 35 | 5 |
| 2 | 12 | 9 | 10 | 3 |
| 11 | 13 | 6 | 10 | 5 |
| 9 | 14 | 6 | 10 | 1 |
| 16 | 15 | 6 | 35 | 3 |
| 5 | 16 | 3 | 35 | 1 |
| 14 | 17 | 6 | 35 | 3 |

**Supplementary Table 2.** Screening of LAB isolates for biosurfactant production

| Sample | Oil displacement  (Zone of displacement) | Emulsification index at 1hr  (%age) | Emulsification index after 48hr  (%age) | Drop collapse  (change in shape from spherical to flat) |
| --- | --- | --- | --- | --- |
| **LAB-BS1** | **1.05 ± 0.35** | **21.8** | **30.6** | **+** |
| **LAB-BS2** | **1.13 ± 0.29** | **40** | **80.5** | **+** |
| **LAB-BS3** | **0.7 ± 0.26** | **20** | **47** | **+** |
| **LAB-BS4** | **1.01 ± 0.26** | **35** | **61** | **+** |
| **LAB-BS5** | **1.03 ± 0.29** | **42** | **60** | **+** |
| **LAB-BS6** | **1.06 ± 0.21** | **23** | **50** | **+** |
| **LAB-BS7** | **1.07 ± 0.15** | **21** | **37** | **+** |
| **LAB-BS8** | **1.10 ± 0.17** | **16** | **29** | **+** |

**Supplementary Table 3.** FTIR peak analysis

| **PEAKS (cm^-1^ )** | **FUNCTIONAL GROUPS** |
| --- | --- |
| 3048 | =CH stretching |
| 2950 |  |
| 1631 | C-H stretching of CH_3_ |
| 1580 |  |
| 1449 |  |
| 1403 | Amide I bond (C=O stretching) |
| 1242 |  |
| 1076 | CH_3_ bending and phosphate |
| 874 |  |

**Supplementary Table 4.** Box-Behnken Design matrix including emulsification index as the response

| **Std.** | **Run** | **Factor 1**  **A: pH** | **Factor 2**  **B: Temperature**  **(°C)** | **Factor 3**  **C: Orange peel concentration**  **(%)** | **Response**  **Emulsification index (%)** |
| --- | --- | --- | --- | --- | --- |
| 10 | 1 | 6 | 60 | 1 | 28 |
| 8 | 2 | 9 | 35 | 5 | 50 |
| 17 | 3 | 6 | 35 | 3 | 65 |
| 13 | 4 | 6 | 35 | 3 | 63 |
| 3 | 5 | 3 | 60 | 3 | 25 |
| 4 | 6 | 9 | 60 | 3 | 23 |
| 1 | 7 | 3 | 10 | 3 | 11 |
| 6 | 8 | 9 | 35 | 1 | 48 |
| 12 | 9 | 6 | 60 | 5 | 30 |
| 15 | 10 | 6 | 35 | 3 | 63 |
| 7 | 11 | 3 | 35 | 5 | 55 |
| 2 | 12 | 9 | 10 | 3 | 17 |
| 11 | 13 | 6 | 10 | 5 | 19 |
| 9 | 14 | 6 | 10 | 1 | 15 |
| 16 | 15 | 6 | 35 | 3 | 67 |
| 5 | 16 | 3 | 35 | 1 | 33 |
| 14 | 17 | 6 | 35 | 3 | 63 |

**Supplementary table 5.** ANOVA of fitted equation of emulsification index

| **Std Dev.** | 2.92 | **R-squared** | 0.9909 |
| --- | --- | --- | --- |
| **Mean** | 39.71 | **Adj R-squared** | 0.9792 |
| **C.V.%** | 7.36 | **Pred- square** | 0.8825 |
|  |  | **Adeq precisior** | 24.289 |
